# Advances in the Development of Antiviral Compounds for Rotavirus Infections

**DOI:** 10.1128/mBio.00111-21

**Published:** 2021-05-11

**Authors:** María Julieta Tohmé, Laura Ruth Delgui

**Affiliations:** a IHEM, Universidad Nacional de Cuyo, CONICET, Facultad de Ciencias Médicas, Mendoza, Argentina; b Facultad de Farmacia y Bioquímica, Universidad Juan Agustín Maza, Mendoza, Argentina; c Facultad de Ciencias Exactas y Naturales, Universidad Nacional de Cuyo, Mendoza, Argentina; University of Cambridge; Albert Einstein College of Medicine

**Keywords:** antiviral, lipid droplets, rotavirus

## Abstract

Group A rotaviruses (RVAs) are the major cause of severe acute gastroenteritis (AGE) in children under 5 years of age, annually resulting in nearly 130,000 deaths worldwide. Social conditions in developing countries that contribute to decreased oral rehydration and vaccine efficacy and the lack of approved antiviral drugs position RVA as a global health concern. In this minireview, we present an update in the field of antiviral compounds, mainly in relation to the latest findings in RVA virion structure and the viral replication cycle. In turn, we attempt to provide a perspective on the possible treatments for RVA-associated AGE, with special focus on novel approaches, such as those representing broad-spectrum therapeutic options. In this context, the modulation of host factors, lipid droplets, and the viral polymerase, which is highly conserved among AGE-causing viruses, are analyzed as possible drug targets.

## INTRODUCTION

Approximately 1.3 million deaths are caused by diarrheal diseases among all ages, with the main impact being on children under 5 years of age (1). Fatal gastroenteritis occurs mainly in developing countries, primarily due to poor hygiene conditions and malnutrition, causing about 300,000 deaths/year in sub-Saharan Africa. However, fatality should not be neglected in high-income countries, where approximately 700 deaths occurred in 2015 (2). Group A rotavirus (RVA) is the most frequent diarrhea-causative agent and continues to be the leading cause of diarrhea-associated mortality among children younger than 5 years, responsible for nearly 130,000 deaths worldwide in 2016 (3). In the small intestine, sodium transport and glucose transport are coupled, which means that the latter also promotes the absorption of solutes and water; therefore, the clinical management of RV-associated diarrhea and dehydration in children is based primarily on the administration of oral rehydration solutions (ORS) (4). ORS are balanced mixtures of glucose and electrolytes that stimulate water absorption to prevent dehydration. Although ORS are effective, their formulation still needs optimization (5, 6).

To decrease the prevalence of diarrhea-associated mortality, the World Health Organization (WHO) recommended the inclusion of RVA vaccines in all national immunization programs of the European region and the Americas in 2006, and this recommendation was extended to all regions worldwide in 2009 (7). The positive impact of RVA vaccination in public health has been demonstrated. However, the incidence and mortality rate of RV infection in high- and low-income locations continue to be quite different from each other. This scenario strongly supports the necessity of antiviral compounds to decrease the number of child deaths as a consequence of acute gastroenteritis (AGE) dehydration.

RV is a segmented double-stranded RNA (dsRNA) virus of the *Reoviridae* family (8). Under an electron microscope, RV is observed as a nonenveloped icosahedral triple-layered particle (TLP) of ∼100 nm in diameter that resembles a wheel (Latin *rota*) (9). RV has an ∼18,500-bp segmented genome with 11 dsRNA molecules that encode six structural proteins (viroplasm 1 [VP1] to -4, VP6, and VP7) and five or six nonstructural proteins depending on the strain (NSP1 to -5/6) (10, 11). Each of the 11 dsRNA segments is associated with one copy of the RNA-dependent RNA polymerase (RdRp) VP1 and the RNA-capping enzyme VP3 (12, 13). Each of the 11 complexes is located beneath each of the 12 5-fold symmetr*y* axes of the innermost layer (14, 15). The innermost icosahedral shell is formed by 60 dimers of the VP2 protein organized in a T=1 lattice, which is an architectural hallmark among nearly all dsRNA viruses and which, once assembled, will never be disassembled during the viral cycle; it constitutes the core or single-layered particle (SLP) of RV (8). The SLP is surrounded by an ∼15-nm-thick icosahedral T=13 capsid made up of 260 pear-shaped VP6 trimers to form the double-layered particle (DLP) (15, 16). In addition to being highly abundant and conserved, VP6 proteins have been shown to be both antigenic and immunogenic (17–21). The DLP is the RV transcriptional machinery that produces capped, nonpolyadenylated, positive-sense, single-stranded RNA (+ssRNA) molecules, capable of initiating an infectious cycle when released into the host cell cytoplasm (22, 23). The third layer is formed by the outer capsid proteins VP4 and VP7. With the same icosahedral triangulation (T=13) as the virion second layer, the glycoprotein VP7 forms 260 calcium-stabilized trimers that sit atop of each VP6 trimer (24). The VP7 layer constitutes the shell from which VP4 spikes protrude. Three copies of VP4 from each viral spike are anchored in the depressions formed in the center of VP7 hexamers (25). This third, outermost layer of the TLP contains the main viral antigens mediating viral entry into the host cell (26).

In this review, an update in the field of antiviral compounds is presented, mainly in relation to the latest findings in RVA virion structure and the infectious cycle. The possible treatments for RVA-caused AGE are also presented.

## BIOCHEMICAL FEATURES OF THE RV INFECTIOUS CYCLE AND POTENTIAL TARGETS FOR THE DEVELOPMENT OF ANTIVIRAL COMPOUNDS AGAINST RV INFECTION

**FIG 1:**
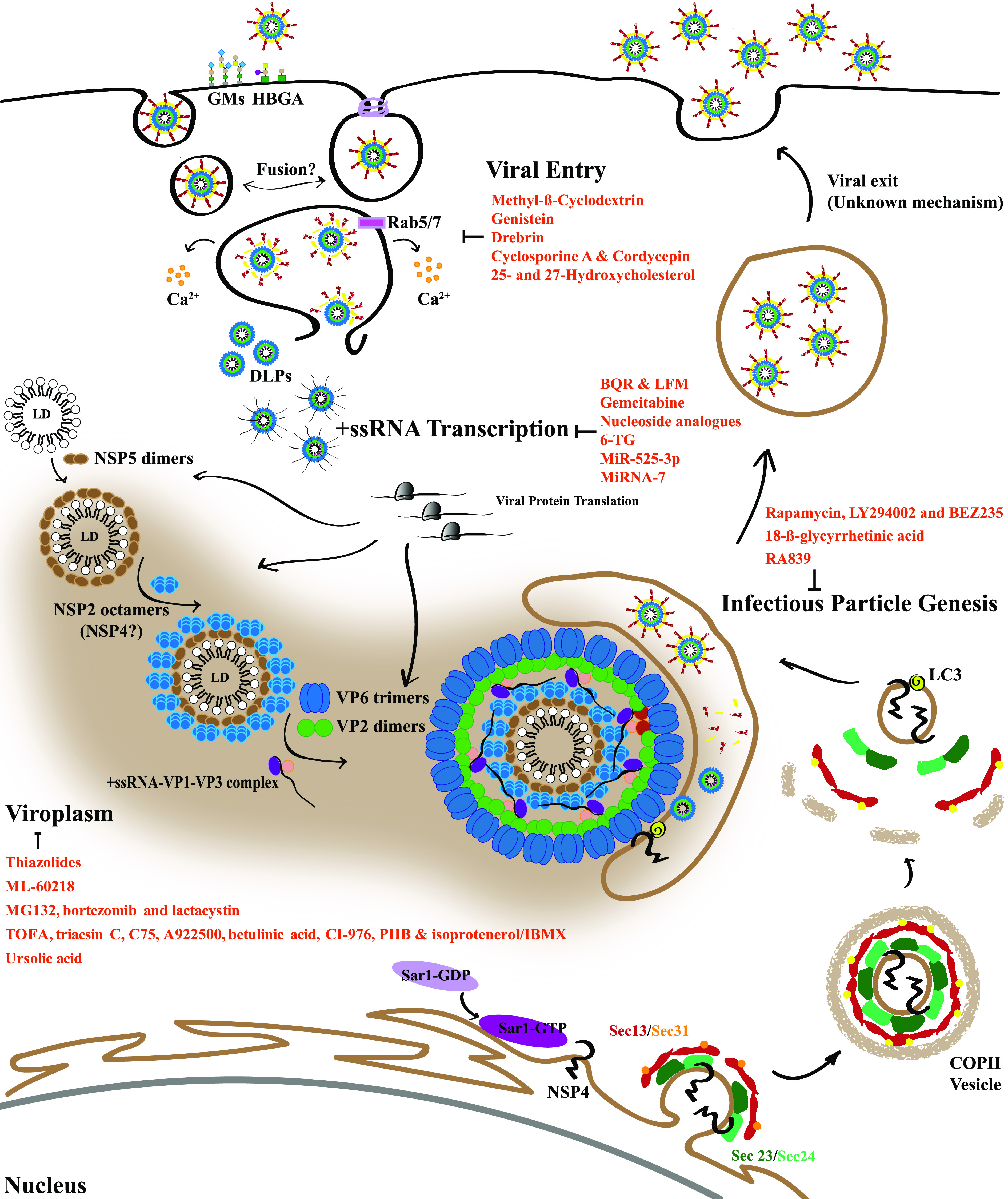
RV life cycle. The first step of the replication cycle consists of the interaction of the infectious particle with glycan attachment molecules. Then, the two models for RV entry are depicted. (Left) Engulfment of the virus in a tight-fitting membrane directed by viral components (38). (Right) Clathrin-dependent (or clathrin-independent) endocytosis, depending on strain. Once in the cytosol, homotypic fusion may occur for RV to finally reach a compartment where calcium ions are extruded from the vesicle to the cytoplasm, producing a progressive decrease of the calcium concentration and leading to the disassembly of the VP7/VP4 layer. Once devoid of the VP7 layer, the VP4 spike is able to perform the fold-back transition, leading to the vesicle bilayer burst and the DLP release into the cytoplasm to initiate viral +ssRNA transcription. After the translation of viral proteins, NSP5 and NSP2 form viroplasms (VPs), where morphogenesis occurs. VPs are large, membrane-less, electron-dense cytoplasmic structures. NSP2, in turn, associates with precore complexes (consisting of VP1, VP3, and segmental +ssRNA), VP2, and VP6 to give rise to immature DLP particles, which interact with NSP4 inserted into the ER. NSP4 is synthesized as an ER transmembrane protein and interacts with Sec24 to be incorporated into COPII vesicles that are released into the cytoplasm. GDP/GTP exchange on Sar1 induces the insertion of Sar1 into the ER and the subsequent recruitment of Sec23/Sec24. Sar1 interacts with Sec23, which allows Sec24 to bind the cargo protein, NSP4, and concentrates these proteins into the nascent COPII vesicle. LC3 interacts with NSP4 and inserts into the NSP4-containing, COPII-derived membranes. The LC3/NSP4-containing membranes traffic to VPs. DLPs produced in the VP interact with NSP4, which triggers particle budding through the LC3/NSP4-containing membranes and infectious particle assembly. The exit of TLPs is by cell lysis or budding (polarized cells).

The RV infectious cycle is exquisitely synchronized with an ordered, stepwise disassembly and assembly of the viral particle layers. Updated data regarding RV life cycle, including the antiviral compounds described in this revision, have been incorporated into the model depicted in Fig. 1. The drug inhibitor class, the mechanism of action, and the stage of development for each compound are summarized in Table 1.

**TABLE 1:** Antiviral compounds for rotavirus infections included in this minireview^*a*^

Antiviral compound(s)	Class(es) of inhibitor(s)	Mechanism(s) of action (reference[s])	Stage of development
Methyl-β-cyclodextrin	Pharmacological cholesterol-sequestering agent	Dispersion of cholesterol rafts and inhibition of RV receptor-mediated endocytosis (34, 35)	Investigational *in vitro* studies
Genistein	Flavonoid, tyrosine kinase inhibitor	Inhibition of integrin phosphorylation, reducing RV binding affinity to integrins and entry (37)	Investigational *in vitro* studies
Drebrin	Cytoskeleton-associated protein	Interaction with VP4 protein and its C-terminal VP5* fragment, impairing RV cell entry (41)	Investigational *in vitro* studies; also tested in human primary enteroids and in an *in vivo* model (C57BL/6 mice)
Cyclosporine (CsA) and cordycepin	Cyclic peptide and adenosine analog, respectively	Increase in the expression of type I interferon (47, 48), promoting antiviral activity	Investigational *in vitro* studies, also tested in a mouse model (BALB/c infant mice); CsA is a well-known and widely used immunosuppressive agent
25- and 27-hydroxycholesterol	Oxysterols	Sequestering of cholesterol in late endosomes, impeding the DLPs from reaching the cytosol (54)	Investigational *in vitro* studies
Brequinar (BQR) and leflunomide (LFM)	DHODH inhibitors	Reducing the host cell nucleoside pool and interfering with viral transcription and replication (63)	Investigational *in vitro* studies, tested in human primary enteroids; both compounds are well-known immunosuppressive agents
Gemcitabine	Cytidine analog	Inhibition of pyrimidine biosynthesis and reduction in the host cell nucleoside pool, interfering with viral transcription and replication (66)	Investigational *in vitro* studies, tested in human primary enteroids; gemcitabine can be found in the “safe-in-man broad-spectrum antiviral agents’ library” and is widely used for cancer treatment
2′-C-methyl nucleoside analogs	Nucleoside analogs	Inhibition of viral RdRps (69)	Investigational *in vitro* studies
6-Thioguanine (6-TG)	Thio- analog of guanine	GTP-Rac1 inhibitor (71)	Investigational *in vitro* studies, tested in human enteroids; 6-TG has been clinically used since the 1950s as an anticancer and immune‐suppressive agent
MiR-525-3p	Cellular microRNA	Binding the 3′ UTR of RV NSP1 and increasing the concn of interferon and cytokines (74)	Investigational *in vitro* studies
miRNA-7	Cellular microRNA	Targeting the 11th RV gene that encodes NSP5, affecting VP formation (75)	Investigational *in vitro* studies, tested in a mouse model (BALB/c infant mice)
Nitazoxanide and tizoxanide	Thiazolides	Hampering NSP5-NSP2 interactions (88–90)	Clinical trial for RV treatment; clinically approved anti-infective treatment
ML-60218	Indazole sulfonamide	Disrupting assembled VPs and hampering the formation of new ones; structural damage on VP6 (97)	Investigational *in vitro* studies
MG132, bortezomib, and lactacystin	Tripeptide aldehyde, dipeptidylboronate, and antibiotic, respectively	Proteasome inhibitors; hampering the formation of VPs (100); altering VP1 localization (101)	Investigational *in vitro* studies
TOFA, triacsin C, C75, A922500, betulinic acid, CI-976, PHB, and isoproterenol/IBMX	Pharmacological enzyme inhibitors of the lipid metabolism pathway	Modulation of LD biogenesis and degradation (reviewed in reference 102)	Investigational *in vitro* studies
Ursolic acid	Triterpenoid	Modulation of LD metabolism (?) (106)	Investigational *in vitro* studies
Rapamycin, LY294002, and BEZ235	Macrolide, morpholine-containing chemical compound, and imidazoquinoline derivative, respectively	mTOR and PI3K inhibitors, respectively; activation of the autophagy cascade (125)	Investigational *in vitro* studies, tested in human enteroids; rapamycin is an FDA-approved drug for transplant patients
18-β-Glycyrrhetinic acid	Aglycone	Modulation of the PI3K/Akt pathway; antiviral activity (127, 128)	Investigational *in vitro* studies, tested in a mouse model (C57BL/6 male mice)
RA839	Small molecule	Activation of the Nfr2/ARE pathway, modulating the redox stress response (132)	Investigational *in vitro* studies

a Abbreviations: RV, rotavirus; LE, late endosomes; DLPs, double-layer particles; DHODH, dihydroorotate dehydrogenase; RdRp, RNA-dependent RNA polymerase; VPs, viroplasms; LD, lipid droplet; Nrf2, nuclear factor 2; ARE, antioxidant response elements; mTOR, mechanistic target of rapamycin; PI3K, phosphatidylinositol 3-kinase; Akt, protein kinase B.

## RV ENTRY INHIBITORS

The viral entry to the host cell is a multistep process occurring after viral attachment, endocytosis, and uncoating to allow the DLPs to reach the cytoplasm for +ssRNA transcription. RV entry is orchestrated by the continuous interaction of VP4 and VP7 with host cell molecules. *In vivo*, RV infects primarily mature enterocytes, but to become fully infectious, TLPs must be cleaved by trypsin-like proteases present in the intestinal lumen (27). This activation step cleaves VP4 to produce two main fragments, the N-terminal VP8* (at the tip of spikes) and the larger C-terminal VP5*.

There is a consensus that the initial virus attachment occurs through the interaction of VP8* with epithelial glycans (10, 28, 29). After that, and depending on the strain, RV interacts with integrins and the heat shock cognate (hsc) protein 70, which have been found to be associated with cell cholesterol-enriched membrane lipid microdomains (10, 28, 30–32). Also, it has been described that the presence of fatty acids enhances RV infectivity at a postattachment step for the SA11 RV strain (33). Using the bovine RV strain CH12, Cui et al. demonstrated that the pretreatment of cells with methyl-β-cyclodextrin (MβCD), a cholesterol-sequestering drug, decreased the levels of viral mRNA, VP4 accumulation, and RV progeny yield. These authors observed that the adsorption of viral particles to the cell membrane was not affected, and no effect was observed when the drug was added after the entry step. Therefore, the authors concluded that MβCD accomplished its antiviral effect by dispersing the cholesterol rafts, thus avoiding receptor-mediated endocytosis (34). Similar results were obtained by Dou et al. with the porcine RV strain DN30209 (35). Integrins are also a target for small-molecule inhibitors. Taking into account that the activity of integrins is controlled by phosphorylation events (36), López et al. treated MA104 cells with the tyrosine-kinase (TK) inhibitor genistein to find a dose- and strain-dependent decrease of RV infectivity. They showed that the anti-RV activity may be attributed to an impairment of RV attachment. Simian SA11 and bovine RF strains were the most sensitive to the drug; the bovine UK, rhesus RRV, neuraminidase-resistant nar3, porcine YM, and human Wa strains were slightly affected, while the porcine TRF-41 strain was insensitive to genistein. Since the less affected strains were those that do not use integrins to invade the host cells, the authors hypothesized that the inhibition of the integrin phosphorylation mediated by TK might be responsible for the reduction of the RV binding affinity to integrins. Even though genistein was found to exert a dramatic reduction of RV infectivity, the drug was highly cytotoxic; therefore, it could not be used as an anti-RV treatment (37).

After the RV attachment to the cell surface, TLPs are internalized into the cytoplasm by receptor-mediated endocytosis (10, 32, 38). Several endocytic routes have been reported to be involved in RV entry, depending on the viral strain (recently reviewed in reference 39). Drebrin, a cytoskeletal protein involved in the stabilization of actin filaments, inhibits endocytosis (40). Li et al. observed that this protein interacts with VP4 and its C-terminal VP5* fragment within the TLP, impairing RV entry at a postattachment stage. Taking into account these biological effects and the fact that actin plays a role in the viral cycle, the authors proposed that the development of drebrin agonists may be a potential broad-spectrum antiviral strategy (41).

Independently of the entry route, all RV strains seem to be incorporated into early endosomes (EEs), since their infectivity depends on the activities of the well-known EE functional regulators Rab5 and EEA1 and the ESCRT (endosomal sorting complex required for transport) machinery (42, 43). On the other hand, the infectivity of several RV strains tested so far depends on the expression of Rab7, suggesting that these viruses travel farther inside the cell to reach late endosomes and behave as late-penetration (LP) viruses (44). The decrease of intraluminal calcium levels promotes the disassembly of the VP7 layer, and soon (typically within 10 min), the outer layer proteins are lost and transcriptionally active DLPs enter the cytosol (23). Infected cells then activate innate immunity mechanisms to reach an antiviral status through the synthesis and secretion of high levels of interferon (IFN) in response to the presence of viral RNA (45). RV has been demonstrated to evade this immune attack by inhibiting the production of IFN (46). Therefore, maintaining high IFN levels in infected cells may be an effective anti-RV strategy. Cyclosporine (CsA), a well-known immunosuppressant agent, has been evaluated as an anti-RV agent. CsA is known to increase the expression of type I IFN both *in vitro* and *in vivo*, causing a significant decrease in RV titers (47). Similar results were found with cordycepin, an adenosine analogue that induces a type I IFN response within the cells (48). Oxysterols are a family of cholesterol oxidation derivatives that exert a wide variety of biochemical effects, one of them being the inhibition of viral replication (49). The bulk of the studies on the antiviral activity of oxysterols has been focused on 25-hydroxycholesterol (25HC), which emerged as a wide-spectrum antiviral molecule against a large number of viruses *in vitro* (49). IFN is known to induce a marked increase in the levels of 25HC in macrophages and dendritic cells. It is an essential cytokine involved in the establishment of the cellular antiviral state through the upregulation of hundreds of interferon-stimulated genes (ISGs). Thus, 25HC can be considered an IFN effector (50). *Ch25* (IFN-inducible cholesterol 25-hydrolxylase) is an antiviral ISG whose product converts cholesterol to 25HC, directly linking the innate antiviral response elicited by IFN to the sterol metabolic pathways. Importantly, the *Ch25* antiviral role against the emerging severe acute respiratory syndrome coronavirus 2 (SARS-CoV-2) has recently been described (51, 52). Civra et al. first tested 25HC and 27HC against the human Wa RV strain and other nonenveloped viruses, observing marked antiviral activities for these compounds (53). Later studies of Civra et al. aimed at ascertaining the anti-RV mechanism of 25HC and 27HC included the WI61, HRV 408, HRV 248, and DS-1 RV strains and demonstrated marked antiviral activities for these compounds (53). All these strains are LP viruses, which require Rab7 to enter the cytosol. The authors showed that the anti-RV effect was mediated by the sequestering of cholesterol in late endosomes (LEs), preventing DLP exit from the LEs to reach the cytosol (54).

## RV RNA TRANSCRIPTION AND GENOME REPLICATION INHIBITORS

The loss of the outermost layer and the arrival of DLPs to the cytosol promote the activation of VP1 and VP3 inside the DLP, allowing viral RNA transcription and the production of +ssRNA (10, 55, 56). The X-ray crystallographic structure of VP1 revealed that the RV RNA-dependent RNA polymerase (RdRp) domain lies within a cage-like sequence with two access channels to accommodate the template RNA and the nucleoside triphosphate substrates and two channels for the exit of ssRNAs in the transcription function and of dsRNA in the replication function. This unique fingers, palm, and thumb organization of RdRp VP1 is shared only with the mammalian orthoreovirus RdRp, λ3 (57–59). Two recent *in situ* studies on RdRp structures have shed light on the transcription and replication mechanisms mediated by VP1 (60, 61). For this step, VP1 requires the biosynthesis of nucleosides by the host cell (62). Chen et al. tested the anti-RV effect of brequinar (BQR) and leflunomide (LFM), two inhibitors of dihydroorotate dehydrogenase, which is an enzyme involved in the biosynthesis of pyrimidines. The authors described the anti-RV effect both *in vitro* and in human enteroids, observing a reduction in viral protein synthesis and a decrease in RV yields for the simian SA11 RV strain and the human clinical isolate 2011K. Based on these observations, the authors claimed that the antiviral effect of BQR and LFM could not be attributed to the strain but to a reduction in the host cell nucleoside pool. The concentrations of both compounds needed to exert the antiviral activity were much lower than the doses used for the treatment of other infections, thus achieving high antiviral efficacy with few side effects (63). On the other hand, gemcitabine is a cytidine analog that inhibits the pyrimidine biosynthesis that is used as an anticancer agent (64). Previous studies showed that gemcitabine is an antienterovirus agent (65). Thus, Chen et al. evaluated the effect of gemcitabine on the rhesus RRV strain and five clinical isolates *in vitro* using Caco2 and MA104 cells and enteroids. Gemcitabine exerted a significant inhibition of RV replication, as evidenced by a decrease in the viral yield, a decrease in the accumulation of viral proteins, and a higher degree of conservation of organoid morphology. Thus, the authors proposed gemcitabine as a feasible anti-RV treatment, mainly in infections of patients with cancer (66). Importantly, the antiviral effect of gemcitabine has already been described for other RNA viruses (67, 68); therefore, the possible use of gemcitabine as a broad-spectrum antiviral cannot be ruled out.

Despite the unique organization of the RV VP1 RdRp domains, its functioning closely resembles that of other viral RdRps (59). Thus, the inhibition of the viral polymerase is a promising antiviral target, not only against RV but also against several related RNA viruses. In this context, Van Dycke et al. studied the effect of four analogs of 2′-C-methyl nucleoside (2′-C-methylguanosine, 2′-C-methyladenosine, 2′-C-methylcytidine, and 7-deaza-2′-C-methyladenosine) against the main RNA AGE-causing viruses, i.e., RV, norovirus, and sapovirus, to find a significant antiviral effect against the three of them. All nucleoside analogues studied proved to be effective anti-RV agents at noncytotoxic concentrations, as evidenced by a decreased accumulation of viral RNA and VP formation, a reduced cytopathic effect, and lower viral yields (69). The nucleoside analogs have been proposed as broad-spectrum antivirals against AGE-causing viruses (70). The clinical symptoms of all viral AGE are very similar; therefore, the availability of a broad-spectrum treatment might allow early treatment initiation, i.e., before virus identification. Finally, 6-thioguanine (6-TG), a thio-analog of guanine widely used as immunosuppressive drug in transplant patients, was tested by Yin et al. (71) in RV infections. The authors demonstrated that 6-TG promoted a decrease in RV +ssRNA levels and viral protein synthesis. The potent anti-RV effect of 6-TG was due to the inhibition of Rac1. Rac1 is a member of the Rho GTPases that is involved in multiple biological processes (72). The role of GTP-Rac1 in RV infection is not clear, but it seems to be necessary for RV replication. This hypothesis was further confirmed when NSC23766, another GTP-Rac1 inhibitor, was demonstrated to have anti-RV properties similar to those of 6-TG (71). It is noteworthy that the anti-RV effect of 6-TG was tested in culture cells and in human organoids using the simian SA11 RV strain and clinical strains to obtain the same results in all cases. Although further research is needed, these authors proposed 6-TG as a promising anti-RV treatment.

MicroRNAs (miRNAs) are cellular short RNA molecules that regulate gene expression by controlling mRNA transcription and stability. The antiviral immunity mediated by IFN also involves the overexpression of multiple miRNAs (73). Among them, MiR-525-3p and miRNA-7 have been studied for their anti-RV properties. MiR-525-3p binds the 3′ untranslated region of RV NSP1, decreasing its expression, and the accumulation of MiR-525-3p is sufficient to inhibit the RV cycle by increasing the concentration of IFN and other cytokines produced by infected cells. Importantly, its anti-RV effect seems not to be strain dependent, since human Wa, simian RRV, and simian SA11 RV strains were effectively inhibited (74). miRNA-7, whose expression is regulated by the host cell, has been described to exert antiviral effects by targeting viral genes or host pathways hijacked by RV. Zhou et al. showed that miRNA-7 is significantly upregulated in RV-infected cells in a multiplicity of infection (MOI)-dependent, strain-independent manner. The wild-type ZTR-68 and ZTR-18, simian SA11, human S2, and porcine Gottfried RV strains were tested. The authors determined that miRNA-7 targets RV gene 11, which encodes NSP5, thereby affecting VP formation and ultimately inhibiting the production of a new RV progeny. The anti-RV effect exerted by miRNA-7 was observed both *in vitro* and *in vivo* (75).

## VP BIOGENESIS AND STABILIZATION INHIBITORS

Replication and packaging of the viral genome into the progeny DLPs take place in specialized, large, membrane-less, electron-dense cytoplasmic inclusions called viroplasms (VPs). NSP5, the main VP protein, together with NSP2 (76), VP2 (77), and lipid droplets (LDs) (78, 79), is crucial for VP formation. The coexpression of NSP5 with either NSP2 or VP2, in the absence of other viral structures, leads to the formation of cytosolic inclusions, named VP-like structures (VLS), which are morphologically similar to VPs but are unable to produce viral progeny (76, 77, 80–82). By reverse-genetics approaches, a phosphorylation-dependent mechanism for VP formation in which the phosphorylation of both NSP2 and NSP5 was required for them to interact and give rise to VPs was demonstrated (83–85). Thiazolides are a big family of widely used anti-infective compounds. The use of thiazolides, particularly nitazoxanide, has been licensed in the United States for the treatment of several AGE-causing agents (86, 87). Rossignol and El-Gohary (88) evaluated its anti-RV effect in a randomized, placebo-controlled, double-blind clinical trial conducted in the Cairo University Children’s Hospital to evaluate pediatric patients. A similar trial was conducted simultaneously in the Benha University Hospital and at the Alexandria University Hospital to evaluate adolescent to adult patients. In all cases, the administration of nitazoxanide significantly reduced the duration of RV AGE, compared to that in control groups (88, 89). Then, La Frazia et al. studied the mechanism of nitazoxanide and its active metabolite, tizoxanide, in an *in vitro* RV model. The authors observed that the treatment of cells with the compounds at 0 to 3 h after RV infection caused the highest reduction in VP size without affecting either viral attachment or viral entry or protein synthesis. Finally, they demonstrated that thiazolides hamper the NSP5-NSP2 interaction, concluding that thiazolides disturb VP formation and stabilization, leading to reduced RV yields (90). The use of nitazoxanide as anti-RV therapy may be a feasible option since it is an approved drug and clinical trials in humans have been conducted to test its effectiveness (91, 92).

Several other RV proteins are involved in VP formation. Buttafuoco et al. recently studied the role of NSP5-VP2 interaction in VLS formation. The authors identified the interacting domains on both proteins and showed that a mutant version of VP2, which is unable to interact with and promote NSP5 phosphorylation, also impedes the VLS formation induced by NSP5-VP2 or NSP5-NSP2 coexpression (93). Aside from these nonstructural proteins, VP1, VP3, VP6, and NSP4 constitute the VPs (94–96). VPs play a key role in the maintenance of the RV infection; therefore, any compound affecting either their synthesis or their structure can be considered a good candidate for the treatment of AGE. One of these compounds is the small molecule called ML-60218 (ML). Eichwald et al. demonstrated that ML affects the formation of VPs and disrupts the assembled ones. Besides, ML-60218-treated DLPs exhibited structural damage that could be attributed to an impairment in the interaction among VP6 trimers that affected their transcriptional activity. However, the authors could not postulate this compound as a possible anti-RV treatment because of its high cytotoxicity and low solubility (97).

The ubiquitin-proteasome system has been described to be modulated by some viruses during infection (98). The proteasome is a complex system that degrades ubiquitinated, altered proteins (99). Contin et al. tested the proteasome inhibitors MG132, bortezomib, and lactacystin in RV infections. These compounds induced an arrest of VP formation, leading to a significant reduction of RV yields of the simian SA11, porcine OSU, and bovine RF strains *in vitro*. The authors observed that the main effect was achieved at early time points of infection but after RV attachment and entry, while no effect was observed on assembled VPs. Thus, they hypothesized that an active proteasome may be necessary to degrade a host protein or proteins that hamper the formation of VPs at early time points of RV infection (100). On the other hand, López et al. also tested the effects of the proteasome inhibitors MG132, lactacystin, and proteasome inhibitor I on the RRV strain. The authors evidenced a significant reduction in RV yields, with MG132 having the strongest effect. These results suggested that the anti-RV effect of MG132 might be partially due to a reduction in the free-amino acid pool, which is necessary for the cellular and viral biosynthetic machinery, and by the accumulation of ubiquitinated proteins. They also observed a reduction in RV replication that might be explained by the presence of smaller VPs and an altered distribution of VP1 (101).

Among the cellular factors, lipid droplets (LDs) are ER-derived intracellular organelles for neutral lipid storage that are required for VP formation. The formation of complexes between VPs and LDs is crucial for RV replication, since compounds disrupting LDs significantly decrease the numbers and sizes of VPs and the RV yield (78, 102). In a recent study, Suárez et al. proposed a model of the nanometric-scale organization of VPs (103). The authors showed that the viral components in the VPs form porous circumferences that are arranged as quite discrete concentric layers. Interestingly, this model proposes the existence of a hollow center inside the first layer formed by NSP5, which might be complemented with a previous observation demonstrating the role of LDs (102). In this context, several inhibitors of lipogenic enzymes have proved to interfere with RV infection. Thus, triacsin C was demonstrated to reduce VP size and number with the corresponding reduction in the viral yield (78, 104). Likewise, tall oil fatty acid (TOFA), alone or in combination with C75, reduces both the infectivity of progeny virus and viral dsRNA production in a time- and dose-dependent manner (105). Reduced viral yields were also observed in RV-infected cells treated with A922500, betulinic acid, CI-976, or polyhydroxybutyrate (PHB) (104). On the other hand, targeting the dispersion of LDs with isoproterenol and isobutylmethylxanthine (IBMX) in RV-infected cells resulted in a reduced number and size of VPs, a decreased production of viral dsRNA, and a 20- to 50-fold-lower yield of infectious progeny (78). In our laboratory, we showed that ursolic acid (UA), a natural pentacyclic triterpenoid, is a potent anti-RV compound *in vitro*. UA significantly decreased RV yields at noncytotoxic concentrations, and its anti-RV effect was maintained when the RV MOI was increased. UA affected the early stages of the RV replication cycle, as evidenced by a significant reduction in the number and size of VPs, the level of viral proteins, and RV yields (106). Since UA is a hypolipidemic agent, we can hypothesize that the anti-RV activity exerted by UA *in vitro* is mediated by the decrease in the availability of LDs for the virus to build the VPs. Due to its multiple biological properties, UA has been extensively studied over the last years (107, 108). Its low bioavailability is the main disadvantage; however, several chemical modifications have successfully been introduced to overcome this obstacle, while none of them has yet been proved to treat RV infection. UA is an FDA-approved drug, representing an attractive candidate for the treatment of viral infections in humans (109, 110).

Within the VPs, NSP2 sorts the RNA complex containing VP1, VP3, and viral +ssRNA. This complex engages VP2 for SLP assembly, while VP1 within newly formed SLP directs the dsRNA synthesis using +ssRNAs as the templates (60, 78, 111, 112). Within the RVA 3′ untranslated region (3′ UTR), Ren et al. (113) identified ATP5B, a core subunit of the mitochondrial ATP synthase, which binds to the consensus region of the 3′ UTR. During RV infection, ATP5B colocalizes with the viral RNA and the VP. The small interfering RNA (siRNA)-mediated genetic depletion of ATP5B reduced the production of infectious viral progeny, with significant alteration of intracellular viral RNA levels and RNA translation. Moreover, the treatment of conventional cell cultures and human enteroids with a panel of small-molecule pharmacological inhibitors of the ATP synthase diminished RV yields, indicating that ATP5B positively regulates RV genome assembly and particle formation (113).

## RV MORPHOGENESIS INHIBITORS

RV morphogenesis starts with the packaging of genome segments in newly formed viral cores, followed by the addition of a second layer of VP6 trimers to form DLPs, which subsequently bud into endoplasmic reticulum (ER) membranes for TLP assembly, although the specific mechanism for the exit of DLPs from VPs remains unknown. The TLP assembly is a unique morphogenetic process that requires NSP4, which is synthesized as an ER transmembrane glycoprotein and with which the glycoprotein VP7 associates within the luminal ER membrane (114–117). The C-terminal cytoplasmic domain of NSP4 hooks the DLPs through binding to VP6, triggering the budding of the DLP through the NSP4-containing membranes, where the particles become transiently enveloped (118). The transient lipid envelope is then removed by an unknown mechanism, and the outer capsid proteins VP7 and VP4 are assembled onto the particle, giving rise to the mature infectious TLP. The intracellular membranes used for particle budding were long thought to be those of the ER because NSP4 is synthesized as an ER transmembrane protein. However, a recent study has shown that NSP4 exits the ER by hijacking ER exit sites in coat complex protein II (COPII) vesicles and that the NSP4-containing COPII vesicles are encountered by the autophagy machinery in the cytoplasm; together, the COPII vesicles and the autophagy machinery mediate the trafficking of NSP4 to VPs. It has been described that NSP4 interacts with Sec24 to be incorporated into COPII vesicles that are released into the cytoplasm. GDP/GTP exchange on Sar1 induces the insertion of Sar1 into the ER and the subsequent recruitment of Sec23/Sec24. Sar1 interacts with Sec23, which allows Sec24 to bind the cargo protein NSP4 and concentrates these proteins into the nascent COPII vesicle. LC3 interacts with NSP4 and inserts itself into the NSP4-containing, COPII-derived membranes. The LC3/NSP4-containing membranes traffic to VPs. These findings support a change in the paradigm as regards membrane origin for RV morphogenesis (119). Although the innate immune response is crucial in viral infections (45), the role of autophagy, an innate immune mechanism, in RV infection is still controversial (120–122).

Many immunosuppressive agents have been tested as possible anti-RV drugs. In this sense, rapamycin is an FDA-approved immunosuppressant used to prevent allograft rejection because of its capacity to inhibit the mammalian target of rapamycin (mTOR), leading to the activation of autophagy (123). Autophagy is modulated by the phosphatidylinositol 3 kinase (PI3K)-protein kinase B (Akt)-mTOR pathway, in which the ultimate phosphorylation/activation of mTOR inhibits the autophagy cascade by the activation of 4E-binding protein 1 (4E-BP1) (124). Yin et al. observed that the use of rapamycin inhibits RV infection by activating the autophagy cascade. These authors also observed that the PI3K inhibitor LY294002 and the PI3K-mTOR inhibitor BEZ235, as well as silencing the mTOR protein expression, effectively reduced RV replication. The authors proposed rapamycin as a feasible anti-RV treatment, especially in transplant patients (125). 18-β-Glycyrrhetinic acid (18βGRA) is an aglycone and the active metabolite of glycyrrhizin, which is present in the licorice root (126). Hardy et al. (127) and Hendricks et al. (128) observed that 18βGRA presents anti-RV activity both *in vitro* and *in vivo*. The antiviral mechanism is not totally clear, but the authors hypothesized that the effects of 18βGRA might be due to its capacity to modulate the PI3K/Akt pathway, as previously demonstrated by Kao et al. (129, 130). The induction of the synthesis of chemokines and the expression of chemokine receptor genes facilitate the recruitment of B cell populations to the gut mucosa, then ameliorating RV-caused diarrhea (127–130).

The cell metabolism produces multiple reactive oxygen species (ROS). As these molecules are harmful when accumulated in great amounts, cells have developed multiple mechanisms to maintain metabolic homeostasis (131). Among them, nuclear factor 2 (Nrf2) is a transcription factor that targets multiple antioxidant response element (ARE) genes involved in the restoring of the reduced cellular environment. Under basal conditions, Nrf2 interacts with the cytosolic Keap1 protein, which prevents Nrf2 translocation to the nucleus. The RV infection is known to cause ER stress, with the subsequent release of ROS from the mitochondria and the induction of the unfolded protein response (UPR) (131). RA839 is a small molecule that specifically binds Keap1, causing Nfr2 to translocate to the nucleus. The treatment of cell cultures with RA839 resulted in an increased expression of antioxidant genes. Patra et al. have demonstrated that RA839 effectively inhibits RV replication, as evidenced by a decrease in the number of VPs, a lower degree of accumulation of structural RV proteins, and lower viral yields. Interestingly, RA839 exerts a potent anti-RV activity mediated by the activation of the Nfr2/ARE pathway at noncytotoxic concentrations *in vitro.* The authors hypothesized that the anti-RV effect of RA839 might be due to the modulation of the redox stress response (132). Antioxidants such as *N*-acetyl cysteine and some anti-inflammatory drugs have also been described as effective treatments in RV-induced AGE, highlighting the importance of the stress response against RV infections (131).

## RV RELEASE INHIBITORS

The final step of the RV life cycle is the TLP release from the infected cell. It has traditionally been thought that RVs are released from nonpolarized cells through a lytic process, with the loss of cell integrity (133–135). Studies using differentiated Caco-2 cells have shown that new virions are almost exclusively released from the apical pole (136–138). Recent studies have additionally demonstrated a crucial role of the actin cytoskeleton in efficient RV exit (139–142). Trejo-Cerro et al. used jasplakinolide, a well-known actin cytoskeleton inhibitor, to show a loss of the preferential apical release of RV from MA104 cells, suggesting a major role for actin in virus release (142). The authors showed that the fraction of extracellular virus was small compared to that of virus that remains associated with the cell; however, based on the notion that this egress mechanism seems to operate in nonpolarized as well as polarized cells, the authors claimed that the actin-dependent viral release might have an important role in the dissemination of the virions *in vitro* and probably *in vivo* (142).

## CONCLUSIONS

Despite the availability of four oral, live, attenuated anti-RV vaccines, RV remains one of the main causes of morbidity and mortality in young children. Several authors have demonstrated the antiviral activities of multiple compounds disrupting the RV replication cycle at different stages (Table 1). Only nitazoxanide has been evaluated in a human double-blind placebo trial in which a significant amelioration of RV AGE was observed. None of the studied compounds so far is currently used as an effective anti-RV treatment.

The host response to viral infections has been an important object of study to find antiviral targets and to develop drugs or gene silencers as potential anti-RV therapies. Some metabolic pathways are highly conserved among cell lines, and since these pathways are used by many viruses, they might serve as targets for broad-spectrum antiviral treatments, as is the case for lipid metabolism. The host provision of LDs is indispensable to establishing a productive RV infection. Then, the use of lipid modulators is an attractive therapeutic approach and constitutes our rational base to study the potential anti-RV effect of UA. Members of the *Flaviviridae* family, namely, dengue virus, hepatitis C virus, West Nile virus, and Zika virus, interact with LDs to usurp the host lipid metabolism to carry out their own viral replication and pathogenesis (143). Therefore, compounds interfering with LD metabolism might be used as extended-spectrum antiviral drugs, including those against viruses infecting humans.

## References

[1] Wang H, Naghavi M, Allen C, Barber RM, Carter A, Casey DC, Charlson FJ, Chen AZ, Coates MM, Coggeshall M, Dandona L, Dicker DJ, Erskine HE, Haagsma JA, Fitzmaurice C, Foreman K, Forouzanfar MH, Fraser MS, Fullman N, Goldberg EM, Graetz N, Haagsma JA, Hay SI, Huynh C, Johnson CO, Kassebaum NJ, Kulikoff XR, Kutz M, Kyu HH, Larson HJ, Leung J, Lim SS, Lind M, Lozano R, Marquez N, Mikesell J, Mokdad AH, Mooney MD, Nguyen G, Nsoesie E, Pigott DM, Pinho C, Roth GA, Sandar L, Silpakit N, Sligar A, Sorensen RJD, Stanaway J, Steiner C, Teeple S, et al. 2016. Global, regional, and national life expectancy, all-cause mortality, and cause-specific mortality for 249 causes of death, 1980–2015: a systematic analysis for the Global Burden of Disease Study 2015. Lancet 388:1459–1544. doi:10.1016/S0140-6736(16)31012-1. 27733281 PMC5388903

[2] Troeger C, Forouzanfar M, Rao PC, Khalil I, Brown A, Reiner RC, Fullman N, Thompson RL, Abajobir A, Ahmed M, Alemayohu MA, Alvis-Guzman N, Amare AT, Antonio CA, Asayesh H, Avokpaho E, Awasthi A, Bacha U, Barac A, Betsue BD, Beyene AS, Boneya DJ, Malta DC, Dandona L, Dandona R, Dubey M, Eshrati B, Fitchett JRA, Gebrehiwot TT, Hailu GB, Horino M, Hotez PJ, Jibat T, Jonas JB, Kasaeian A, Kissoon N, Kotloff K, Koyanagi A, Kumar GA, Rai RK, Lal A, El Razek HMA, Mengistie MA, Moe C, Patton G, Platts-Mills JA, Qorbani M, Ram U, Roba HS, Sanabria J, Sartorius B, Sawhney M, et al. 2017. Estimates of global, regional, and national morbidity, mortality, and aetiologies of diarrhoeal diseases: a systematic analysis for the Global Burden of Disease Study 2015. Lancet Infect Dis 17:909–948. doi:10.1016/S1473-3099(17)30276-1. 28579426 PMC5589208

[3] Troeger C, Khalil IA, Rao PC, Cao S, Blacker BF, Ahmed T, Armah G, Bines JE, Brewer TG, Colombara DV, Kang G, Kirkpatrick BD, Kirkwood CD, Mwenda JM, Parashar UD, Petri WA, Riddle MS, Steele AD, Thompson RL, Walson JL, Sanders JW, Mokdad AH, Murray CJL, Hay SI, Reiner RC. 2018. Rotavirus vaccination and the global burden of rotavirus diarrhea among children younger than 5 years. JAMA Pediatr 172:958–965. doi:10.1001/jamapediatrics.2018.1960. 30105384 PMC6233802

[4] Nalin DR, Mata L, Vargas W, Rosa Loria A, Levine MM, De Cespedes C, Lizano C, Simhon A, Mohs E. 1978. Comparison of sucrose with glucose in oral therapy of infant diarrhoea. Lancet 312:277–279. doi:10.1016/S0140-6736(78)91686-0. 79080

[5] Buccigrossi V, Lo Vecchio A, Bruzzese E, Russo C, Marano A, Terranova S, Cioffi V, Guarino A. 2020. Potency of oral rehydration solution in inducing fluid absorption is related to glucose concentration. Sci Rep 10:7803. doi:10.1038/s41598-020-64818-3. 32385331 PMC7210290

[6] Kaunitz JD. 2020. Oral defense: how oral rehydration solutions revolutionized the treatment of toxigenic diarrhea. Dig Dis Sci 65:345–348. doi:10.1007/s10620-019-06023-5. 31900719 PMC7193728

[7] World Health Organization. 2009. Rotavirus vaccines: an update. Wkly Epidemiol Rec 84:533–540. 20034143

[8] Attoui H, Becnel J, Belaganahalli S, Bergoin M, Brussaard CP, Chappell JD, Ciarlet M, del Vas M, Dermody TS, Dormitzer PR, Duncan R, Fang Q, Graham R, Guglielmi KM, Harding RM, Hillman B, Makkay A, Marzachì AC, Matthijnssens J, Mertens PPC, Milne RG, Mohd Jaafar F, Mori H, Noordeloos AA, Omura T, Patton JT, Rao S, Maan M, Stoltz D, Suzuki N, Upadhyaya NM, Wei C, Zhou H. 2012. Family-Reoviridae, p 541–637. *In* King AMQ, Adams MJ, Carstens EB, Lefkowitz EJ (ed), Virus taxonomy: classification and nomenclature of viruses. Ninth report of the International Committee on Taxonomy of Viruses. Elsevier Academic Press, San Diego, CA.

[9] Prasad BVV, Wang GJ, Clerx JPM, Chiu W. 1988. Three-dimensional structure of rotavirus. J Mol Biol 199:269–275. doi:10.1016/0022-2836(88)90313-0. 2832610

[10] Estes MK, Greenberg HB. 2013. Rotaviruses, p 1347–1401. In Fields virology, 6th ed. Wolters Kluwer Health, Philadelphia, PA.

[11] Hu L, Crawford SE, Hyser JM, Estes MK, Prasad BVV. 2012. Rotavirus non-structural proteins: structure and function. Curr Opin Virol 2:380–388. doi:10.1016/j.coviro.2012.06.003. 22789743 PMC3422752

[12] Patton JT, Chen D. 1999. RNA-binding and capping activities of proteins in rotavirus open cores. J Virol 73:1382–1391. doi:10.1128/JVI.73.2.1382-1391.1999. 9882343 PMC103962

[13] Periz J, Celma C, Jing B, Pinkney JNM, Roy P, Kapanidis AN. 2013. Rotavirus mRNAs are released by transcript-specific channels in the double-layered viral capsid. Proc Natl Acad Sci U S A 110:12042–12047. doi:10.1073/pnas.1220345110. 23818620 PMC3718169

[14] Estrozi LF, Settembre EC, Goret G, McClain B, Zhang X, Chen JZ, Grigorieff N, Harrison SC. 2013. Location of the dsRNA-dependent polymerase, VP1, in rotavirus particles. J Mol Biol 425:124–132. doi:10.1016/j.jmb.2012.10.011. 23089332 PMC3540981

[15] McClain B, Settembre E, Temple BRS, Bellamy AR, Harrison SC. 2010. X-ray crystal structure of the rotavirus inner capsid particle at 3.8 Å resolution. JMol Biol 397:587–599. doi:10.1016/j.jmb.2010.01.055. 20122940 PMC2860780

[16] Zhang X, Settembre E, Xu C, Dormitzer PR, Bellamy R, Harrison SC, Grigorieff N. 2008. Near-atomic resolution using electron cryomicroscopy and single-particle reconstruction. Proc Natl Acad Sci U S A 105:1867–1872. doi:10.1073/pnas.0711623105. 18238898 PMC2542862

[17] Tang B, Gilbert JM, Matsui SM, Greenberg HB. 1997. Comparison of the rotavirus gene 6 from different species by sequence analysis and localization of subgroup-specific epitopes using site-directed mutagenesis. Virology 237:89–96. doi:10.1006/viro.1997.8762. 9344910

[18] Svensson L, Sheshberadaran H, Vene S, Norrby E, Grandien M, Wadell G. 1987. Serum antibody responses to individual viral polypeptides in human rotavirus infections. J Gen Virol 68:643–651. doi:10.1099/0022-1317-68-3-643. 3029295

[19] Svensson L, Sheshberadaran H, Vesikari T, Norrby E, Wadell G. 1987. Immune response to rotavirus polypeptides after vaccination with heterologous rotavirus vaccines (RIT 4237, RRV-1). J Gen Virol 68:1993–1999. doi:10.1099/0022-1317-68-7-1993. 3037019

[20] Ishida S, Feng N, Tang B, Gilbert JM, Greenberg HB. 1996. Quantification of systemic and local immune responses to individual rotavirus proteins during rotavirus infection in mice. J Clin Microbiol 34:1694–1700. doi:10.1128/JCM.34.7.1694-1700.1996. 8784572 PMC229097

[21] Colomina J, Gil MT, Codoñer P, Buesa J. 1998. Viral proteins VP2, VP6, and NSP2 are strongly precipitated by serum and fecal antibodies from children with rotavirus symptomatic infection. J Med Virol 56:58–65. doi:10.1002/(SICI)1096-9071(199809)56:1<58::AID-JMV10>3.0.CO;2-S. 9700634

[22] Bass DM, Baylor MR, Chen C, Mackow EM, Bremont M, Greenberg HB. 1992. Liposome-mediated transfection of intact viral particles reveals that plasma membrane penetration determines permissivity of tissue culture cells to rotavirus. J Clin Invest 90:2313–2320. doi:10.1172/JCI116119. 1334974 PMC443384

[23] Salgado EN, Upadhyayula S, Harrison SC. 2017. Single-particle detection of transcription following rotavirus entry. J Virol 91:e00651-17. doi:10.1128/JVI.00651-17. 28701394 PMC5571246

[24] Aoki ST, Settembre EC, Trask SD, Greenberg HB, Harrison SC, Dormitzer PR. 2009. Structure of rotavirus outer-layer protein VP7 bound with a neutralizing Fab. Science 324:1444–1447. doi:10.1126/science.1170481. 19520960 PMC2995306

[25] Settembre EC, Chen JZ, Dormitzer PR, Grigorieff N, Harrison SC. 2011. Atomic model of an infectious rotavirus particle. EMBO J 30:408–416. doi:10.1038/emboj.2010.322. 21157433 PMC3025467

[26] López S, Arias CF. 2017. Rotavirus biology, p 19–43. In Ludert J, Pujol F, Arbiza J (ed), Human virology in Latin America. Springer, Cham, Switzerland.

[27] Graham DY, Estes MK. 1980. Proteolytic enhancement of rotavirus infectivity: biologic mechanisms. Virology 101:432–439. doi:10.1016/0042-6822(80)90456-0. 6244698

[28] López S, Arias CF. 2004. Multistep entry of rotavirus into cells: a Versaillesque dance. Trends Microbiol 12:271–278. doi:10.1016/j.tim.2004.04.003. 15165605

[29] Lopez S, Arias CF. 2006. Early steps in rotavirus cell entry. Curr Top Microbiol Immunol 309:39–66. doi:10.1007/3-540-30773-7_2. 16909896

[30] Fukudome K, Yoshie O, Konno T. 1989. Comparison of human, simian, and bovine rotaviruses for requirement of sialic acid in hemagglutination and cell adsorption. Virology 172:196–205. doi:10.1016/0042-6822(89)90121-9. 2549710

[31] Baker M, Prasad BVV. 2010. Rotavirus cell entry. Curr Top Microbiol Immunol 343:121–148. doi:10.1007/82_2010_34. 20397068

[32] Desselberger U. 2014. Rotaviruses. Virus Res 190:75–96. doi:10.1016/j.virusres.2014.06.016. 25016036

[33] Superti F, Marziano ML, Donelli G, Marchetti M, Seganti L. 1995. Enhancement of rotavirus infectivity by saturated fatty acids. Comp Immunol Microbiol Infect Dis 18:129–135. doi:10.1016/0147-9571(95)98854-B. 7621669

[34] Cui J, Fu X, Xie J, Gao M, Hong M, Chen Y, Su S, Li S. 2014. Critical role of cellular cholesterol in bovine rotavirus infection. Virol J 11:98. doi:10.1186/1743-422X-11-98. 24884772 PMC4053397

[35] Dou X, Li Y, Han J, Zarlenga DS, Zhu W, Ren X, Dong N, Li X, Li G. 2018. Cholesterol of lipid rafts is a key determinant for entry and post-entry control of porcine rotavirus infection. BMC Vet Res 14:1–12. doi:10.1186/s12917-018-1366-7. 29433482 PMC5809846

[36] Gahmberg CG, Grönholm M, Uotila LM. 2014. Regulation of integrin activity by phosphorylation. Adv Exp Med Biol 819:85–96. doi:10.1007/978-94-017-9153-3_6. 25023169

[37] López T, López S, Arias CF. 2015. The tyrosine kinase inhibitor genistein induces the detachment of rotavirus particles from the cell surface. Virus Res 210:141–148. doi:10.1016/j.virusres.2015.07.020. 26216271

[38] Abdelhakim AH, Salgado EN, Fu X, Pasham M, Nicastro D, Kirchhausen T, Harrison SC. 2014. Structural correlates of rotavirus cell entry. PLoS Pathog 10:e1004355. doi:10.1371/journal.ppat.1004355. 25211455 PMC4161437

[39] Arias CF, Silva-Ayala D, López S. 2015. Rotavirus entry: a deep journey into the cell with several exits. J Virol 89:890–893. doi:10.1128/JVI.01787-14. 25378490 PMC4300671

[40] Hayashi K. 2017. Cell shape change by drebrin. Adv Exp Med Biol 1006:83–101. doi:10.1007/978-4-431-56550-5_6. 28865016

[41] Li B, Ding S, Feng N, Mooney N, Ooi YS, Ren L, Diep J, Kelly MR, Yasukawa LL, Patton JT, Yamazaki H, Shirao T, Jackson PK, Greenberg HB. 2017. Drebrin restricts rotavirus entry by inhibiting dynamin-mediated endocytosis. Proc Natl Acad Sci U S A 114:E3642–E3651. doi:10.1073/pnas.1619266114. 28416666 PMC5422808

[42] Silva-Ayala D, López T, Gutiérrez M, Perrimon N, López S, Arias CF, Estes MK. 2013. Genome-wide RNAi screen reveals a role for the ESCRT complex in rotavirus cell entry. Proc Natl Acad Sci U S A 110:10270–10275. doi:10.1073/pnas.1304932110. 23733942 PMC3690850

[43] Wolf M, Deal EM, Greenberg HB. 2012. Rhesus rotavirus trafficking during entry into MA104 cells is restricted to the early endosome compartment. J Virol 86:4009–4013. doi:10.1128/JVI.06667-11. 22278225 PMC3302545

[44] Diaz-Salinas MA, Silva-Ayala D, Lopez S, Arias CF. 2014. Rotaviruses reach late endosomes and require the cation-dependent mannose-6-phosphate receptor and the activity of cathepsin proteases to enter the cell. J Virol 88:4389–4402. doi:10.1128/JVI.03457-13. 24501398 PMC3993738

[45] Katze MG, He Y, Gale M. 2002. Viruses and interferon: a fight for supremacy. Nat Rev Immunol 2:675–687. doi:10.1038/nri888. 12209136

[46] Holloway G, Truong TT, Coulson BS. 2009. Rotavirus antagonizes cellular antiviral responses by inhibiting the nuclear accumulation of STAT1, STAT2, and NF- B. J Virol 83:4942–4951. doi:10.1128/JVI.01450-08. 19244315 PMC2682104

[47] Shen Z, He H, Wu Y, Li J. 2013. Cyclosporin A inhibits rotavirus replication and restores interferon-beta signaling pathway in vitro and in vivo. PLoS One 8:e71815. doi:10.1371/journal.pone.0071815. 23990993 PMC3749198

[48] Chanda SD, Banerjee A. 2015. Cordycepin an adenosine analogue executes anti rotaviral effect by stimulating induction of type I interferon. J Virol Antiviral Res 04:2. doi:10.4172/2324-8955.1000138.

[49] Lembo D, Cagno V, Civra A, Poli G. 2016. Oxysterols: an emerging class of broad spectrum antiviral effectors. Mol Aspects Med 49:23–30. doi:10.1016/j.mam.2016.04.003. 27086126

[50] Park K, Scott AL. 2010. Cholesterol 25-hydroxylase production by dendritic cells and macrophages is regulated by type I interferons. J Leukoc Biol 88:1081–1087. doi:10.1189/jlb.0610318. 20699362 PMC2996899

[51] Zang R, Case JB, Yutuc E, Ma X, Shen S, Castro MFG, Liu Z, Zeng Q, Zhao H, Son J, Rothlauf PW, Kreutzberger AJB, Hou G, Zhang H, Bose S, Wang X, Vahey MD, Mani K, Griffiths WJ, Kirchhausen T, Fremont DH, Guo H, Diwan A, Wang Y, Diamond MS, Whelan SPJ, Ding S. 2020. Cholesterol 25-hydroxylase suppresses SARS-CoV-2 replication by blocking membrane fusion. Proc Natl Acad Sci U S A 117:32105–32113. doi:10.1073/pnas.2012197117. 33239446 PMC7749331

[52] Wang S, Li W, Hui H, Tiwari SK, Zhang Q, Croker BA, Rawlings S, Smith D, Carlin AF, Rana TM. 2020. Cholesterol 25-hydroxylase inhibits SARS -CoV-2 and other coronaviruses by depleting membrane cholesterol. EMBO J 39:e106057. doi:10.15252/embj.2020106057. 32944968 PMC7537045

[53] Civra A, Cagno V, Donalisio M, Biasi F, Leonarduzzi G, Poli G, Lembo D. 2014. Inhibition of pathogenic non-enveloped viruses by 25-hydroxycholesterol and 27-hydroxycholesterol. Sci Rep 4:7487. doi:10.1038/srep07487. 25501851 PMC4265783

[54] Civra A, Francese R, Gamba P, Testa G, Cagno V, Poli G, Lembo D. 2018. 25-Hydroxycholesterol and 27-hydroxycholesterol inhibit human rotavirus infection by sequestering viral particles into late endosomes. Redox Biol 19:318–330. doi:10.1016/j.redox.2018.09.003. 30212801 PMC6138790

[55] Trask SD, Ogden KM, Patton JT. 2012. Interactions among capsid proteins orchestrate rotavirus particle functions. Curr Opin Virol 2:373–379. doi:10.1016/j.coviro.2012.04.005. 22595300 PMC3422376

[56] Cohen J, Laporte J, Charpilienne A, Scherrer R. 1979. Activation of rotavirus RNA polymerase by calcium chelation. Arch Virol 60:177–186. doi:10.1007/BF01317489. 41504

[57] Tao Y, Farsetta DL, Nibert ML, Harrison SC. 2002. RNA synthesis in a cage—structural studies of reovirus polymerase lambda3. Cell 111:733–745. doi:10.1016/S0092-8674(02)01110-8. 12464184

[58] McDonald SM, Tao YJ, Patton JT. 2009. The ins and outs of four-tunneled Reoviridae RNA-dependent RNA polymerases. Curr Opin Struct Biol 19:775–782. doi:10.1016/j.sbi.2009.10.007. 19914820 PMC2798595

[59] Lu X, McDonald SM, Tortorici MA, Tao YJ, Vasquez-Del Carpio R, Nibert ML, Patton JT, Harrison SC. 2008. Mechanism for coordinated RNA packaging and genome replication by rotavirus polymerase VP1. Structure 16:1678–1688. doi:10.1016/j.str.2008.09.006. 19000820 PMC2602806

[60] Ding K, Celma CC, Zhang X, Chang T, Shen W, Atanasov I, Roy P, Zhou ZH. 2019. In situ structures of rotavirus polymerase in action and mechanism of mRNA transcription and release. Nat Commun 10:2216. doi:10.1038/s41467-019-10236-7. 31101900 PMC6525196

[61] Jenni S, Salgado EN, Herrmann T, Li Z, Grant T, Grigorieff N, Trapani S, Estrozi LF, Harrison SC. 2019. In situ structure of rotavirus VP1 RNA-dependent RNA polymerase. J Mol Biol 431:3124–3138. doi:10.1016/j.jmb.2019.06.016. 31233764 PMC6697194

[62] Chen D, Patton JT. 2000. De novo synthesis of minus strand RNA by the rotavirus RNA polymerase in a cell-free system involves a novel mechanism of initiation. RNA 6:1455–1467. doi:10.1017/S1355838200001187. 11073221 PMC1370016

[63] Chen S, Ding S, Yin Y, Xu L, Li P, Peppelenbosch MP, Pan Q, Wang W. 2019. Suppression of pyrimidine biosynthesis by targeting DHODH enzyme robustly inhibits rotavirus replication. Antiviral Res 167:35–44. doi:10.1016/j.antiviral.2019.04.005. 30974126

[64] Guinan M, Benckendorff C, Smith M, Miller GJ. 2020. Recent advances in the chemical synthesis and evaluation of anticancer nucleoside analogues. Molecules 25:2050. doi:10.3390/molecules25092050. PMC7248840 32354007

[65] Lee K, Eun KD, Jang KS, Kim SJ, Cho S, Kim C. 2017. Gemcitabine, a broad-spectrum antiviral drug, suppresses enterovirus infections through innate immunity induced by the inhibition of pyrimidine biosynthesis and nucleotide depletion. Oncotarget 8:115315–115325. doi:10.18632/oncotarget.23258. 29383162 PMC5777774

[66] Chen S, Wang Y, Li P, Yin Y, Bijvelds M, de Jonge H, Peppelenbosch MP, Kainov D, Pan Q. 2020. Drug screening identifies gemcitabine inhibiting rotavirus through alteration of pyrimidine nucleotide synthesis pathway. Antiviral Res 180:104823. doi:10.1016/j.antiviral.2020.104823. 32485209 PMC7261112

[67] Beran RKF, Sharma R, Corsa AC, Tian Y, Golde J, Lundgaard G, Delaney WE, IV, Zhong W, Greenstein AE. 2012. Cellular growth kinetics distinguish a cyclophilin inhibitor from an HSP90 inhibitor as a selective inhibitor of hepatitis C virus. PLoS One 7:e30286. doi:10.1371/journal.pone.0030286. 22347373 PMC3275588

[68] Dyall J, Coleman CM, Hart BJ, Venkataraman T, Holbrook MR, Kindrachuk J, Johnson RF, Olinger GG, Jahrling PB, Laidlaw M, Johansen LM, Lear-Rooney CM, Glass PJ, Hensley LE, Frieman MB. 2014. Repurposing of clinically developed drugs for treatment of Middle East respiratory syndrome coronavirus infection. Antimicrob Agents Chemother 58:4885–4893. doi:10.1128/AAC.03036-14. 24841273 PMC4136000

[69] Van Dycke J, Arnoldi F, Papa G, Vandepoele J, Burrone OR, Mastrangelo E, Tarantino D, Heylen E, Neyts J, Rocha-Pereira J. 2018. A single nucleoside viral polymerase inhibitor against norovirus, rotavirus, and sapovirus-induced diarrhea. J Infect Dis 218:1753–1758. doi:10.1093/infdis/jiy398. 30085019

[70] Bassetto M, Van Dycke J, Neyts J, Brancale A, Rocha-Pereira J. 2019. Targeting the viral polymerase of diarrhea-causing viruses as a strategy to develop a single broad-spectrum antiviral therapy. Viruses 11:173. doi:10.3390/v11020173. PMC6409847 30791582

[71] Yin Y, Chen S, Hakim MS, Wang W, Xu L, Dang W, Qu C, Verhaar AP, Su J, Fuhler GM, Peppelenbosch MP, Pan Q. 2018. 6-Thioguanine inhibits rotavirus replication through suppression of Rac1 GDP/GTP cycling. Antiviral Res 156:92–101. doi:10.1016/j.antiviral.2018.06.011. 29920300 PMC7113846

[72] Nguyen LK, Kholodenko BN, von Kriegsheim A. 2018. Rac1 and RhoA: networks, loops and bistability. Small GTPases 9:316–321. doi:10.1080/21541248.2016.1224399. 27533896 PMC5997137

[73] Gebert LFR, MacRae IJ. 2019. Regulation of microRNA function in animals. Nat Rev Mol Cell Biol 20:21–37. doi:10.1038/s41580-018-0045-7. 30108335 PMC6546304

[74] Tian Z, Zhang J, He H, Li J, Wu Y, Shen Z. 2017. MiR-525-3p mediates antiviral defense to rotavirus infection by targeting nonstructural protein 1. Biochim Biophys Acta 1863:3212–3225. doi:10.1016/j.bbadis.2017.09.003. 28890396

[75] Zhou Y, Chen L, Du J, Hu X, Xie Y, Wu J, Lin X, Yin N, Sun M, Li H. 2020. MicroRNA-7 inhibits rotavirus replication by targeting viral NSP5 in vivo and in vitro. Viruses 12:209–217. doi:10.3390/v12020209. PMC7077326 32069901

[76] Fabbretti E, Afrikanova I, Vascotto F, Burrone OR. 1999. Two non-structural rotavirus proteins, NSP2 and NSP5, form viroplasm-like structures in vivo. J Gen Virol 80:333–339. doi:10.1099/0022-1317-80-2-333. 10073692

[77] Contin R, Arnoldi F, Campagna M, Burrone OR. 2010. Rotavirus NSP5 orchestrates recruitment of viroplasmic proteins. J Gen Virol 91:1782–1793. doi:10.1099/vir.0.019133-0. 20200190

[78] Cheung W, Gill M, Esposito A, Kaminski CF, Courousse N, Chwetzoff S, Trugnan G, Keshavan N, Lever A, Desselberger U. 2010. Rotaviruses associate with cellular lipid droplet components to replicate in viroplasms, and compounds disrupting or blocking lipid droplets inhibit viroplasm formation and viral replication. J Virol 84:6782–6798. doi:10.1128/JVI.01757-09. 20335253 PMC2903253

[79] Saxena K, Blutt SE, Ettayebi K, Zeng X-L, Broughman JR, Crawford SE, Karandikar UC, Sastri NP, Conner ME, Opekun AR, Graham DY, Qureshi W, Sherman V, Foulke-Abel J, In J, Kovbasnjuk O, Zachos NC, Donowitz M, Estes MK. 2016. Human intestinal enteroids: a new model to study human rotavirus infection, host restriction, and pathophysiology. J Virol 90:43–56. doi:10.1128/JVI.01930-15. 26446608 PMC4702582

[80] Eichwald C, Vascotto F, Fabbretti E, Burrone OR. 2002. Rotavirus NSP5: mapping phosphorylation sites and kinase activation and viroplasm localization domains. J Virol 76:3461–3470. doi:10.1128/JVI.76.7.3461-3470.2002. 11884570 PMC136013

[81] Eichwald C, Rodriguez JF, Burrone OR. 2004. Characterization of rotavirus NSP2/NSP5 interactions and the dynamics of viroplasm formation. J Gen Virol 85:625–634. doi:10.1099/vir.0.19611-0. 14993647

[82] Eichwald C, Arnoldi F, Laimbacher AS, Schraner EM, Fraefel C, Wild P, Burrone OR, Ackermann M. 2012. Rotavirus viroplasm fusion and perinuclear localization are dynamic processes requiring stabilized microtubules. PLoS One 7:e47947. doi:10.1371/journal.pone.0047947. 23110139 PMC3479128

[83] Papa G, Venditti L, Arnoldi F, Schraner EM, Potgieter C, Borodavka A, Eichwald C, Burrone OR. 2019. Recombinant rotaviruses rescued by reverse genetics reveal the role of NSP5 hyperphosphorylation in the assembly of viral factories. J Virol 94:e01110-19. doi:10.1128/JVI.01110-19. 31619556 PMC6912106

[84] Criglar JM, Hu L, Crawford SE, Hyser JM, Broughman JR, Prasad BVV, Estes MK. 2014. A novel form of rotavirus NSP2 and phosphorylation-dependent NSP2-NSP5 interactions are associated with viroplasm assembly. J Virol 88:786–798. doi:10.1128/JVI.03022-13. 24198401 PMC3911676

[85] Criglar JM, Crawford SE, Estes MK. 2021. Plasmid-based reverse genetics for probing phosphorylation-dependent viroplasm formation in rotaviruses. Virus Res 291:198193. doi:10.1016/j.virusres.2020.198193. 33053412 PMC8820476

[86] Fox LM, Saravolatz LD. 2005. Nitazoxanide: a new thiazolide antiparasitic agent. Clin Infect Dis 40:1173–1180. doi:10.1086/428839. 15791519

[87] Pankuch GA, Appelbaum PC. 2006. Activities of tizoxanide and nitazoxanide compared to those of five other thiazolides and three other agents against anaerobic species. Antimicrobial Agents Chemother 50:1112–1117. doi:10.1128/AAC.50.3.1112-1117.2006. PMC1426457 16495282

[88] Rossignol JF, El-Gohary YM. 2006. Nitazoxanide in the treatment of viral gastroenteritis: a randomized double-blind placebo-controlled clinical trial. Aliment Pharmacol Ther 24:1423–1430. doi:10.1111/j.1365-2036.2006.03128.x. 17081163

[89] Rossignol J-F, Abu-Zekry M, Hussein A, Santoro G. 2006. Effect of nitazoxanide for treatment of severe rotavirus diarrhoea: randomised double-blind placebo-controlled trial. Lancet 368:124–129. doi:10.1016/S0140-6736(06)68852-1. 16829296

[90] La Frazia S, Ciucci A, Arnorldi F, Coira M, Gianferretti P, Angelini M, Belardo G, Burrone OR, Rossignol JF, Santoro MG. 2013. Thiazolides, a new class of antiviral agents effective against rotavirus infection, target viral morphogenesis inhibiting viroplasm formation. J Virol 87:11096–11106. doi:10.1128/JVI.01213-13. 23926336 PMC3807293

[91] Mahapatro S, Mahilary N, Satapathy AK, Das RR. 2017. Nitazoxanide in acute rotavirus diarrhea: a randomized control trial from a developing country. J Trop Med 2017:1–5. doi:10.1155/2017/7942515. PMC5346365 28331496

[92] Rossignol JF. 2014. Nitazoxanide: a first-in-class broad-spectrum antiviral agent. Antiviral Res 110:94–103. doi:10.1016/j.antiviral.2014.07.014. 25108173 PMC7113776

[93] Buttafuoco A, Michaelsen K, Tobler K, Ackermann M, Fraefel C, Eichwald C. 2020. Conserved rotavirus NSP5 and VP2 domains interact and affect viroplasm. J Virol 94:e01965-19. doi:10.1128/JVI.01965-19. 31915278 PMC7081909

[94] Berkova Z, Crawford SE, Trugnan G, Yoshimori T, Morris AP, Estes MK. 2006. Rotavirus NSP4 induces a novel vesicular compartment regulated by calcium and associated with viroplasms. J Virol 80:6061–6071. doi:10.1128/JVI.02167-05. 16731945 PMC1472611

[95] Arnoldi F, Campagna M, Eichwald C, Desselberger U, Burrone OR. 2007. Interaction of rotavirus polymerase VP1 with nonstructural protein NSP5 is stronger than that with NSP2. J Virol 81:2128–2137. doi:10.1128/JVI.01494-06. 17182692 PMC1865955

[96] Pesavento JB, Crawford SE, Estes MK, Venkataram Prasad BV. 2006. Rotavirus proteins: structure and assembly. Curr Top Microbiol Immunol 309:189–219. doi:10.1007/3-540-30773-7_7. 16913048

[97] Eichwald C, De Lorenzo G, Schraner EM, Papa G, Bollati M, Swuec P, de Rosa M, Milani M, Mastrangelo E, Ackermann M, Burrone OR, Arnoldi F. 2018. Identification of a small molecule that compromises the structural integrity of viroplasms and rotavirus double-layered particles. J Virol 92:e01943-17. doi:10.1128/JVI.01943-17. 29142132 PMC5774888

[98] Randow F, Lehner PJ. 2009. Viral avoidance and exploitation of the ubiquitin system. Nat Cell Biol 11:527–534. doi:10.1038/ncb0509-527. 19404332

[99] Nandi D, Tahiliani P, Kumar A, Chandu D. 2006. The ubiquitin-proteasome system. J Biosci 31:137–155. doi:10.1007/BF02705243. 16595883

[100] Contin R, Arnoldi F, Mano M, Burrone OR. 2011. Rotavirus replication requires a functional proteasome for effective assembly of viroplasms. J Virol 85:2781–2792. doi:10.1128/JVI.01631-10. 21228236 PMC3067976

[101] López T, Silva-Ayala D, López S, Arias CF. 2011. Replication of the rotavirus genome requires an active ubiquitin-proteasome system. J Virol 85:11964–11971. doi:10.1128/JVI.05286-11. 21900156 PMC3209302

[102] Crawford SE, Desselberger U. 2016. Lipid droplets form complexes with viroplasms and are crucial for rotavirus replication. Curr Opin Virol 19:11–15. doi:10.1016/j.coviro.2016.05.008. 27341619 PMC5125616

[103] Suárez YG, Martínez JL, Hernández DT, Hernández HO, Pérez-Delgado A, Méndez M, Wood CD, Rendon-Mancha JM, Silva-Ayala D, López S, Guerrero A, Arias CF. 2019. Nanoscale organization of rotavirus replication machineries. Elife 8:e42906. doi:10.7554/eLife.42906. 31343403 PMC6692110

[104] Kim Y, George D, Prior AM, Prasain K, Hao S, Le DD, Hua DH, Chang KO. 2012. Novel triacsin C analogs as potential antivirals against rotavirus infections. Eur J Med Chem 50:311–318. doi:10.1016/j.ejmech.2012.02.010. 22365411 PMC3312795

[105] Gaunt ER, Cheung W, Richards JE, Lever A, Desselberger U. 2013. Inhibition of rotavirus replication by downregulation of fatty acid synthesis. J Gen Virol 94:1310–1317. doi:10.1099/vir.0.050146-0. 23486665

[106] Tohmé MJ, Gimenez MC, Peralta A, Colombo MI, Delgui LR. 2019. Ursolic acid: a novel antiviral compound inhibiting rotavirus infection in vitro. Int J Antimicrob Agents 54:601–609. doi:10.1016/j.ijantimicag.2019.07.015. 31356859

[107] Ramos-Hryb AB, Pazini FL, Kaster MP, Rodrigues ALS. 2017. Therapeutic potential of ursolic acid to manage neurodegenerative and psychiatric diseases. CNS Drugs 31:1029–1041. doi:10.1007/s40263-017-0474-4. 29098660

[108] Yin R, Li T, Tian JX, Xi P, Liu RH. 2018. Ursolic acid, a potential anticancer compound for breast cancer therapy. Crit Rev Food Sci Nutr 58:568–574. doi:10.1080/10408398.2016.1203755. 27469428

[109] Hussain H, Green IR, Ali I, Khan IA, Ali Z, Al-Sadi AM, Ahmed I. 2017. Ursolic acid derivatives for pharmaceutical use: a patent review (2012–2016). Expert Opin Ther Pat 27:1061–1072. doi:10.1080/13543776.2017.1344219. 28637397

[110] Manayi A, Nikan M, Nobakht-Haghighi N, Abdollahi M. 2019. Advances in the anticancer value of the ursolic acid through nanodelivery. Curr Med Chem 25:4866–4875. doi:10.2174/0929867324666170713102918. 28707589

[111] Borodavka A, Dykeman EC, Schrimpf W, Lamb DC. 2017. Protein-mediated RNA folding governs sequence-specific interactions between rotavirus genome segments. Elife 6:e27453. doi:10.7554/eLife.27453. 28922109 PMC5621836

[112] Trask SD, McDonald SM, Patton JT. 2012. Structural insights into the coupling of virion assembly and rotavirus replication. Nat Rev Microbiol 10:165–177. doi:10.1038/nrmicro2673. 22266782 PMC3771686

[113] Ren L, Ding S, Song Y, Li B, Ramanathan M, Co J, Amieva MR, Khavari PA, Greenberg HB. 2019. Profiling of rotavirus 3UTR-binding proteins reveals the ATP synthase subunit ATP5B as a host factor that supports late-stage virus replication. J Biol Chem 294:5993–6006. doi:10.1074/jbc.RA118.006004. 30770472 PMC6463704

[114] Stirzaker SC, Whitfeld PL, Christie DL, Bellamy AR, Both GW. 1987. Processing of rotavirus glycoprotein VP7: implications for the retention of the protein in the endoplasmic reticulum. J Cell Biol 105:2897–2903. doi:10.1083/jcb.105.6.2897. 2826493 PMC2114692

[115] Arias CF, López S, Espejo RT. 1982. Gene protein products of SA11 simian rotavirus genome. J Virol 41:42–50. doi:10.1128/JVI.41.1.42-50.1982. 6283128 PMC256724

[116] Ericson BL, Graham DY, Mason BB, Hanssen HH, Estes MK. 1983. Two types of glycoprotein precursors are produced by the simian rotavirus SA11. Virology 127:320–332. doi:10.1016/0042-6822(83)90147-2. 6306912

[117] Both GW, Siegman LJ, Bellamy AR, Atkinson PH. 1983. Coding assignment and nucleotide sequence of simian rotavirus SA11 gene segment 10: location of glycosylation sites suggests that the signal peptide is not cleaved. J Virol 48:335–339. doi:10.1128/JVI.48.2.335-339.1983. 6312090 PMC255357

[118] López T, Camacho M, Zayas M, Nájera R, Sánchez R, Arias CF, López S, 2005. Silencing the morphogenesis of rotavirus. J Virol 79:184–192. doi:10.1128/JVI.79.1.184-192.2005. 15596814 PMC538724

[119] Crawford SE, Criglar JM, Liu Z, Broughman JR, Estes MK. 2019. COPII vesicle transport is required for rotavirus NSP4 interaction with the autophagy protein LC3 II and trafficking to viroplasms. J Virol 94:e01341-19. doi:10.1128/JVI.01341-19. 31597778 PMC6912103

[120] Tian G, Liang X, Chen D, Mao X, Yu J, Zheng P, He J, Huang Z, Yu B. 2016. Vitamin D3 supplementation alleviates rotavirus infection in pigs and IPEC-J2 cells via regulating the autophagy signaling pathway. J Steroid Biochem Mol Biol 163:157–163. doi:10.1016/j.jsbmb.2016.05.004. 27174720

[121] Mukhopadhyay U, Chanda S, Patra U, Mukherjee A, Rana S, Mukherjee A, Chawla-Sarkar M. 2019. Synchronized orchestration of miR-99b and let-7g positively regulates rotavirus infection by modulating autophagy Sci Rep 9:1318. doi:10.1038/s41598-018-38473-8. 30718795 PMC6362297

[122] Arnoldi F, De Lorenzo G, Mano M, Schraner EM, Wild P, Eichwald C, Burrone OR. 2014. Rotavirus increases levels of lipidated LC3 supporting accumulation of infectious progeny virus without inducing autophagosome formation. PLoS One 9:e95197. doi:10.1371/journal.pone.0095197. 24736649 PMC3988245

[123] Weichhart T, Hengstschläger M, Linke M. 2015. Regulation of innate immune cell function by mTOR. Nat Rev Immunol 15:599–614. doi:10.1038/nri3901. 26403194 PMC6095456

[124] Glick D, Barth S, Macleod KF. 2010. Autophagy: cellular and molecular mechanisms. J Pathol 221:3–12. doi:10.1002/path.2697. 20225336 PMC2990190

[125] Yin Y, Dang W, Zhou X, Xu L, Wang W, Cao W, Chen S, Su J, Cai X, Xiao S, Peppelenbosch MP, Pan Q. 2017. PI3K-Akt-mTOR axis sustains rotavirus infection via the 4E-BP1 mediated autophagy pathway and represents an antiviral target. Virulence 9:83–98. doi:10.1080/21505594.2017.1326443. 28475412 PMC5955461

[126] Hattori M, Sakamoto T, Yamagishi T, Sakamoto K, Konishi K, Kobashi K, Namba T. 1985. Metabolism of glycyrrhizin by human intestinal flora. II. Isolation and characterization of human intestinal bacteria capable of metabolizing glycyrrhizin and related compounds. Chem Pharm Bull 33:210–217. doi:10.1248/cpb.33.210. 4006016

[127] Hardy ME, Hendricks JM, Paulson JM, Faunce NR. 2012. 18Β-glycyrrhetinic acid inhibits rotavirus replication in culture. Virol J 9:96. doi:10.1186/1743-422X-9-96. 22616823 PMC3478227

[128] Hendricks JM, Hoffman C, Pascual DW, Hardy ME. 2012. 18β-Glycyrrhetinic acid delivered orally induces isolated lymphoid follicle maturation at the intestinal mucosa and attenuates rotavirus shedding. PLoS One 7:e49491. doi:10.1371/journal.pone.0049491. 23152913 PMC3496704

[129] Kao TC, Shyu MH, Yen GC. 2010. Glycyrrhizic acid and 18β-glycyrrhetinic acid inhibit inflammation via PI3K/Akt/GSK3β signaling and glucocorticoid receptor activation. J Agric Food Chem 58:8623–8629. doi:10.1021/jf101841r. 20681651

[130] Kao TC, Shyu MH, Yen GC. 2009. Neuroprotective effects of glycyrrhizic acid and 18β-glycyrrhetinic acid in PC12 cells via modulation of the PI3K/Akt pathway. J Agric Food Chem 57:754–761. doi:10.1021/jf802864k. 19105645

[131] Guerrero CA, Acosta O. 2016. Inflammatory and oxidative stress in rotavirus infection. World J Virol 5:38. doi:10.5501/wjv.v5.i2.38. 27175349 PMC4861870

[132] Patra U, Mukhopadhyay U, Sarkar R, Mukherjee A, Chawla-Sarkar M. 2019. RA-839, a selective agonist of Nrf2/ARE pathway, exerts potent anti-rotaviral efficacy in vitro. Antiviral Res 161:53–62. doi:10.1016/j.antiviral.2018.11.009. 30465784

[133] Altenburg BC, Graham DY, Estes MK. 1980. Ultrastructural study of rotavirus replication in cultured cells. J Gen Virol 46:75–85. doi:10.1099/0022-1317-46-1-75. 6243348

[134] Musalem C, Espejo RT. 1985. Release of progeny virus from cells infected with simian rotavirus SA11. J Gen Virol 66:2715–2724. doi:10.1099/0022-1317-66-12-2715. 2999314

[135] McNulty MS, Curran WL, McFerran JB. 1976. The morphogenesis of a cytopathic bovine rotavirus in Madin Darby bovine kidney cells. J Gen Virol 33:503–508. doi:10.1099/0022-1317-33-3-503. 826605

[136] Jourdan N, Maurice M, Delautier D, Quero AM, Servin AL, Trugnan G. 1997. Rotavirus is released from the apical surface of cultured human intestinal cells through nonconventional vesicular transport that bypasses the Golgi apparatus. J Virol 71:8268–8278. doi:10.1128/JVI.71.11.8268-8278.1997. 9343179 PMC192285

[137] Ciarlet M, Crawford SE, Estes MK. 2001. Differential infection of polarized epithelial cell lines by sialic acid-dependent and sialic acid-independent rotavirus strains. J Virol 75:11834–11850. doi:10.1128/JVI.75.23.11834-11850.2001. 11689665 PMC114770

[138] Cevallos Porta D, López S, Arias CF, Isa P. 2016. Polarized rotavirus entry and release from differentiated small intestinal cells. Virology 499:65–71. doi:10.1016/j.virol.2016.09.010. 27639572

[139] Gardet A, Breton M, Fontanges P, Trugnan G, Chwetzoff S. 2006. Rotavirus spike protein VP4 binds to and remodels actin bundles of the epithelial brush border into actin bodies. J Virol 80:3947–3956. doi:10.1128/JVI.80.8.3947-3956.2006. 16571811 PMC1440440

[140] Gardet A, Breton M, Trugnan G, Chwetzoff S. 2007. Role for actin in the polarized release of rotavirus. J Virol 81:4892–4894. doi:10.1128/JVI.02698-06. 17301135 PMC1900189

[141] Condemine W, Eguether T, Couroussé N, Etchebest C, Gardet A, Trugnan G, Chwetzoff S. 2018. The C terminus of rotavirus VP4 protein contains an actin binding domain which requires cooperation with the coiled-coil domain for actin remodeling. J Virol 93:e01598-18. doi:10.1128/JVI.01598-18. 30333172 PMC6288328

[142] Trejo-Cerro O, Eichwald C, Schraner EM, Silva-Ayala D, López S, Arias CF. 2018. Actin-dependent nonlytic rotavirus exit and infectious virus morphogenetic pathway in nonpolarized cells. J Virol 92:e02076-17. doi:10.1128/JVI.02076-17. 29263265 PMC5827380

[143] Martín-Acebes MA, de Oya NJ, Saiz JC. 2019. Lipid metabolism as a source of druggable targets for antiviral discovery against zika and other flaviviruses. Pharmaceuticals 12:97. doi:10.3390/ph12020097. PMC6631711 31234348

